# Modeling the resumption of work and production of enterprises during COVID-19: An SIR-based quantitative framework

**DOI:** 10.3389/fpubh.2022.1066299

**Published:** 2022-12-16

**Authors:** Hongchao Zhao, Zili Huang, Lei Xu, Junqing Tang, Yuang Chen

**Affiliations:** ^1^Department of Trade Economics, Renmin Business School, Renmin University of China, Beijing, China; ^2^School of Science and Engineering, The Chinese University of Hong Kong, Shenzhen, China; ^3^Shenzhen Research Institute of Big Data, Shenzhen, China; ^4^Georgia Tech Shenzhen Institute, Tianjin University, Shenzhen, China; ^5^School of Urban Planning and Design, Shenzhen Graduate School, Peking University, Shenzhen, China

**Keywords:** COVID-19 pandemic, economic loss, industrial enterprises, SIR model, simulation

## Abstract

The ongoing COVID-19 pandemic has evolved beyond being a public health crisis as it has exerted worldwide severe economic impacts, triggering cascading failures in the global industrial network. Although certain powerful enterprises can remain its normal operation during this global shock, what's more likely to happen for the majority, especially those small- and medium-sized firms, is that they are experiencing temporary suspension out of epidemic control requirement, or even permanent closure due to chronic business losses. For those enterprises that sustain the pandemic and only suspend for a relatively short period, they could resume work and production when epidemic control and prevention conditions are satisfied and production and operation are adjusted correspondingly. In this paper, we develop a novel quantitative framework which is based on the classic susceptible-infectious-recovered (SIR) epidemiological model (i.e., the SIR model), containing a set of differential equations to capture such enterprises' reactions in response to COVID-19 over time. We fit our model from the resumption of work and production (RWP) data on industrial enterprises above the designated size (IEDS). By modeling the dynamics of enterprises' reactions, it is feasible to investigate the ratio of enterprises' state of operation at given time. Since enterprises are major economic entities and take responsibility for most output, this study could potentially help policy makers better understand the economic impact caused by the pandemic and could be heuristic for future prevention and resilience-building strategies against suchlike outbreaks of public health crises.

## 1. Introduction

### 1.1. Background information

On 30 January 2020, WHO declared the COVID-19 outbreak a Public Health Emergency of International Concern. Governments worldwide have taken various control and mitigation measures to limit the spread of the virus, which comes at a cost of suspension of work and production nationwide. The COVID-19 pandemic turns out to be not only a public health crisis but also an economic one (i.e., in 2020, a recent investigation carried out by The World Bank showed that the world GDP fell by 3.29% because of COVID-19 impacts). Thus, exploring the economic impact of COVID-19 and related mechanism have become an important research subject. One perspective to study the adverse impact of the pandemic is to investigate what enterprises of various sizes have experienced during and after COVID-19.

By March 2020, China has quickly taken the COVID-19 outbreak under control by implementing stringent measures, such as lockdown, restrictions of human mobility, and public gathering (1, 2). The number of new daily confirmed cases in China peaked on 14 February, 2020 (WHO). By the end of March, most provinces, except Hubei, the epicenter of the first round outbreak, have already lessened restrictions on movement and shifted their focus to reviving the economy. However, effective virus containment measures were carried out at the cost of substantial economic losses. In the first quarter of 2020, China's GDP contracted by 6.8% from the same quarter a year ago; the utilization rate of national industrial capacity was 8.6% lower than the same period of 2019; the total value added of the industrial enterprises above the designated size (IEDS)^1^ went down by 8.4% year on year (3).

There is a high level of interdependency and connectedness among the closure of enterprises, especially for those within one supply chain network. How the state of one enterprise will influence another is, however, not clear. Furthermore, an enterprise can experience several possible operation states during an outbreak, from normal operation, operation suspension to full recovery. To understand how these possible states evolve over time and how fast the evolution are important in that supportive intervention strategies (e.g., those preventing an enterprise from closure) can be designed accordingly. Nevertheless, existing studies have not yet proposed proper method to model the evolving state of an enterprise over time and thus are incapable of providing insights toward the interdependency of enterprise closures. This paper proposes a novel analytical framework to simulate the operation states of enterprises during COVID-19, with the goal to understand how enterprises respond to public health crisis and supportive intervention policy making, concerning post-pandemic resilient recovery of work, and production during COVID-19. Our framework is based on the classic susceptible-infectious-recovered (SIR) epidemiological model, which is wildly used in infectious disease study. The basic logic of SIR model is to classified the population by their infectious status, and use a system of ODEs to simulate the spread of the disease. Based on the SIR model, we classify the business state of each enterprise into four compartments and develop a compartment-based framework to quantify the evolving state of an enterprise over time. Specifically, at each time period, an enterprise will be in one of the following four states: (1) it may remain in operation throughout the epidemic without any suspension or even shut down; (2) it may also be in the state of temporary suspension due to various reasons, such as financial pressure, epidemic control, supply chain disruptions, and more; (3) it can be permanently shut down; and another possible state is that (4) it is currently operating normally but the difference from the first state is that it first experience a suspension and then recover to normal state. For easy exposition, we term these four states as in normal operation, temporary suspension, permanent shutdown, and in recovered operation. Four coupled differential equations, which involve four time-dependent functions, are used to describe the dynamics of the number of enterprises in each class.

We collect data on enterprises resuming production from China's four provinces (Anhui, Hebei, Heilongjiang, and Shandong) to fit the model and calibrate the model by the least square method. Specifically, we collect data about the province-daily-level ratio of IEDS that have resumed production from news announcement and government website. In 2020, Chinese government extended Chinese New Year Holiday, which was supposed to end on January 30, to February 2 due to the epidemic. Most provinces, including Anhui, Hebei, Heilongjiang, and Shandong, officially extended the resumption of work and production (RWP) to February 10^2^. Before official RWP, the ratio of IEDS that have resumed production is only 21.8% in Shandong, and less than 14% in Anhui and Hebei (As for Heilongjiang, the RWP ratio we collect starts from February 17). By mid March, that ratio has increased to over 90% in all four provinces. Although we do not have data on the ratio from a daily basis, we do manage to collect the data on most of the observation days. In terms of data, the relation between COVID-19 and its adverse impact on small and medium-sized enterprises (SME) has been amply investigated in the literature [see (4–6)], while the impact of the epidemic on the IEDS is still an open field of research. This article utilized real data on IEDS to investigate the rate of resumption of work and production during and after a wave of COVID-19 in China.

### 1.2. Literature review

It is widely accepted that there are trade-offs between economic and health outcomes under COVID-19. Abandoning the containment policy too early would avoid a sharp drop in output and employment in the short term, but it would greatly increase mortality and ultimately lead to a decline in social welfare (7).

Since the outbreak of COVID-19 pandemic, there has been a large number of emerging studies devoted to evaluating the consequent economic loss using real data or by simulation. Their focuses range from macroeconomic consequences, such as inflation, unemployment, and exchange rates (8–10), supply chain disruptions (11–14), to influence on financial market (15–17), labor market (18–20), and firms (21–25). During the pandemic, people cut back on consumption and work to reduce the chances of being infected (26).

Studies on China's economic loss under COVID-19 paid special attention to the economic impact of lockdown. Chen et al. (27) found that a 1-month full-scale lockdown causally reduces the truck flows connected to the locked down city and the city's real income in the month by 54%. Cities in lockdown experienced a 34% reduction in the year-on-year growth rate of exports (28). Hubei province, which experienced the most drastic lockdown in China, lost 37% in GDP compared to the counterfactual situation without lockdown; the losses in value added of agriculture, secondary, and service industry are 17%, 46%, and 31%, respectively (29).

The main purpose of our work is to develop a mathematical model to study the evolution of enterprises' operation states, such as suspension and production resumption, under the pandemic outbreak. It is found that, in China, the manufacture sector was scheduled to resume work the earliest (5), and experienced a quick V-shape recovery (30). In February 2020, China's manufacturing Purchasing Manager Index (PMI) was 35.7%. In March, the index has increased by 16.3 percentage points to 52%, 2 percentage points higher than that in January. Labor shortages and the increased operating costs were the main obstacles hindering the manufacturing industry from resuming full production (5). According to Dai et al. (6), 47.6% of employees were unable to return to work in manufacturing enterprises, the highest among all sectors in February 2020. Higher prices of raw material, logistics, and labor were all adding to increases operation costs (27). Besides, enterprises had to pour resources and efforts into disinfection and protective measures, which further led to higher costs and lower efficiency. Using online surveys on small and medium-sized enterprises from Sichuan province, China, Lu et al. (5) found that the manufacturing industry is more likely to face product delivery and supply chain pressures, and less likely to face financial pressures. Gu et al. (4) examined the impact of COVID-19 on firms' activities using daily electricity usage data for 34,040 micro-enterprises in Suzhou city, China in the period from December 31, 2019 to March 31, 2020, via difference-in-differences method. They found that the manufacturing industry incurred the greatest negative impact at the early stage of the pandemic.

In general, our work is also widely related to the literature on the impact of disasters on enterprises. The effects of disasters differ across types of disasters and economic sectors (31). An important research question concerns enterprises' recovery from natural disasters (32–37). The key point is that more efficient firms are less likely to go bankrupt after an earthquake both inside and outside of the affected areas (38), as also found in Cole et al. (39) which explored the birth, life, and death of manufacturing plants after the 1995 Kobe earthquake and shows that the continuing plants experience a temporary increase in productivity, while those most likely to exit are the least productive ones. Apart from productivity, how enterprises perform during disasters will also depend on their position in a supply chain (40), and how well they are prepared and how they respond to the risk (41).

To sum up, it is an important research topic to learn the economic impact of COVID-19. To understand how enterprises are affected, existing studies have mainly focused on conducting surveys or providing empirical evidence. Apart from these methods, mathematical model is a useful but currently rarely used tool. On this basis, our work propose an SIR-based modeling framework to analyze the RWP of enterprises during COVID-19 to enrich our understanding of the impact of the pandemic on enterprises.

### 1.3. Contribution statement

The contributions of this work can be summarized as follows. First, this study borrows ideas from the infectious disease community and novelly applies the SIR model to characterize the evolving state of enterprises affected by the COVID-19 pandemic. The compartments of enterprises are realistically modeled as those remain in operation, temporary suspension, permanent shutdown, and those finally recover to normal state. Second, we conduct a case study of four provinces in China to mimic the actual RWP process during the first wave of COVID-19 outbreak. We find that the resumption rate is the largest in Shandong province, and the smallest in Heilongjiang. Besides, the differences in parameter value among these provinces are reflected in governments' policy intensity to support RWP. Third, we conduct numerical simulations to examine the sensitivity of the model output to changes in parameter value, and provide policy implications on this basis. The simulations results indicate that governments' supportive policies toward enterprises under an epidemic should be primarily focused on limiting bankruptcy and accelerating resumption rate. Policies aimed at depressing suspension rate would have little impact on reducing economic loss to an outbreak.

## 2. The proposed SIR-based quantitative framework

In this section, we propose an SIR-based modeling framework to characterize the evolving states of enterprises' operation under COVID-19. This framework is neat, yet representative, for modeling the real-world situations without complex parameter settings.

The SIR model, proposed by Kermack and McKendrick (42), is widely used to describe the spread of an epidemic virus [e.g., (43–47)]. The basic logic is to divide the population into three compartments: *susceptible* (individuals who are healthy but can contract the disease), *infected* (individuals who have contracted the disease and are infectious), and *recovered* (individuals who have recovered and cannot contract the disease again). An individual is classed in the *susceptible* compartment before contracting the disease. When a susceptible individual enters into contact with an infectious individual and contracts the disease, the susceptible individual moves from the *susceptible* compartment into the *infected* compartment and becomes infectious. Those individuals who recover from the disease gain immunity and will not contract the disease again. At the same time, they leave the *infected* compartment for the *recovered* compartment. Two parameters are used to denote the rate at which individuals move from one compartment to another. The value of epidemiological parameters reflects characteristics of the virus studied, such as infectiousness, recovery time, fatality, and so on. They are also influenced by some aspects of the affected community, such as demographic characteristics, measures taken to mitigate local transmission and so on.

**Figure 1:**
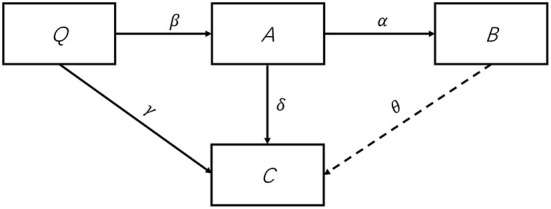
Flowchart of enterprises in a pandemic.

In our framework, the study object is enterprise instead of people. Enterprises are divided into four classes (Figure 1): enterprises that are in normal operation (denoted by compartment *Q*), enterprises that suspend operation (denoted by compartment *A*), enterprises that resume operation after suspension (denoted by compartment *B*), and enterprises that are permanently shut down (denoted by compartment *C*). Before an outbreak occurs, all enterprises are in normal operation (*Q*). Since the onset of the outbreak, they could either suspend operation (*A*) out of epidemic prevention requirements or operation difficulties, or close permanently (*C*). Enterprises move from normal operation to suspension at a rate of β, and to permanent closure at a rate of γ. Then, enterprises in suspension (*A*) would gradually resume operation (*B*) at a rate of α, or move from temporary closure to a permanent one (*C*) if they cannot survive. δ is the rate at which enterprises move from suspension to closure. Specifically, we put all enterprises that have resumed operation into compartment *B*, no matter if they operate at full or partial capacity after work resumption. The transformation from one compartment to another is indicated by a link arrow in Figure 1. It is important to note that, we assume during one wave of an epidemic, enterprises that have resumed operation will not go into suspension again or bankrupt, that is, no link arrow exists from compartment *B* to compartment *A* or *C*. And there is no new enterprises established during the time.

To highlight the time-varying change of these compartments, we formally represent *Q, A, B, C* as *Q*(*t*), *A*(*t*), *B*(*t*), and *C*(*t*), which are functions of time *t*. The total number of enterprises are denoted by *N*, which is assumed constant over the study. The sum of the sizes of these four compartments at each time period *t* satisfies:


(1) $$\begin{array}{l} N=Q\left(t\right)+A\left(t\right)+B\left(t\right)+C\left(t\right) \end{array}$$

To ease exposition, we normalize the constant *N* = 1. And the model is represented by a system of ordinary differential equations (ODEs) as follows:


(2) $$\begin{array}{l} \frac{dQ\left(t\right)}{dt}=-\beta Q-\gamma Q \end{array}$$

(3) $$\begin{array}{l} \frac{dA\left(t\right)}{dt}=\beta Q-\alpha AB-\delta A \end{array}$$

(4) $$\begin{array}{l} \frac{dB\left(t\right)}{dt}=\alpha AB \end{array}$$

(5) $$\begin{array}{l} \frac{dC\left(t\right)}{dt}=\gamma Q+\delta A \end{array}$$

Equations (2), (3), (4), and (5) describe the change of compartments *Q*, *A*, *B*, and *C*, respectively. Notice that the number of enterprises moving from suspension to resumption per unit of time is non-linear. It is not only positively correlated to the number of enterprises in compartment *A*, but also positively correlated to the number of enterprises in compartment *B*. The latter positive correlation is derived based on the observation that, for enterprises to resume operation, they need to make sure the epidemic control and prevention conditions are satisfied and production are correspondingly adjusted. Enterprises that have resumed operation could provide not only knowledge and experience on epidemic control and prevention, but also confidence in operation resumption. Thus, we propose that more enterprises in compartment *B* lead to faster movement of enterprises from compartment *A* to *B*. At the onset of an epidemic, all enterprises are in the class of normal operation, i.e., *Q*(0) = 1, *A*(0) = *B*(0) = *C*(0) = 0. As time proceeds, these enterprises could move to different classes and constitute values of *A*(*t*), *B*(*t*), *C*(*t*) at time *t*.

We next present two special cases of the abovementioned model, which can mimic the realistic situation as well. The main difference of these two special models from the general one is that analytical solutions can be directly derived and thus commercial solver is not required to solve these two models. In the first case, we assume that all enterprises suspend operation at the onset of an epidemic. This could happen if lockdown is imposed immediately. In this case, the model can be reduced to having only three compartments, *A*, *B*, and *C*. It can be simply represented as follows:


(6) $$\begin{array}{l} \frac{dA\left(t\right)}{dt}=-\alpha AB-\delta A \end{array}$$

(7) $$\begin{array}{l} \frac{dB\left(t\right)}{dt}=\alpha AB \end{array}$$

(8) $$\begin{array}{l} \frac{dC\left(t\right)}{dt}=\delta A \end{array}$$

By making assumptions that *A*(*T*_*end*_) = 0, *B*(*T*_*end*_) + *C*(*T*_*end*_) = 1, dividing Equation (7) by Equation (8) we get $\frac{dB}{dC}=\frac{\alpha }{\delta }B$, and thus compartment *B* can be approximately written as an exponential function $B=e^{\frac{\alpha }{\delta }C}-1$. Moreover, since *A*(*t*) + *B*(*t*) + *C*(*t*) = 1, we have $A=1-B-C=2-e^{\frac{\alpha }{\delta }C}-C$. We can also have the function expression of *B*(*t*) and *C*(*t*) by making some assumptions on the function expression of *C*(*t*). In the second case, we assume that there are no enterprises going into permanent closure. That is, the model consists of only two compartments, *A* and *B*. This could happen if the community recover quickly from the epidemic, or enterprises in the study are equipped with abundant supportive policies and well prepared to weather the negative shock. In this case, the model takes the form:


(9) $$\begin{array}{l} A^{\prime }\left(t\right)=-\alpha AB \end{array}$$

(10) $$\begin{array}{l} B^{\prime }\left(t\right)=\alpha AB \end{array}$$

We could derive the analytical representation of compartment *A* as $A=\frac{A\left(T_{k}\right)e^{\alpha \left(T_{k}-t\right)}}{A\left(T_{k}\right)e^{\alpha \left(T_{k}-t\right)}+1-A\left(T_{k}\right)}$, where *A*(*T*_*k*_) is the value of *A* at time *T*_*k*_. By letting *B*(*t*) = 1−*A*(*t*), we end up with the analytical expression of *B*(*t*).

## 3. Data description

The data we use is the province-daily-level ratio of IEDS that have resumed production from four provinces in China, which are Anhui, Hebei, Heilongjiang, and Shandong. In 2020, Chinese New Year Holiday and post-holiday work resumption were both officially extended due to COVID-19. February 10 marked the start of formal RWP in most provinces where the epidemic had been under control. Since then, the RWP ratio among IEDS was often released to make public the progress in RWP. We manually collect the raw data from local governments' press conference and official website^3^ and use it for model calibration purpose.

The population of enterprises here is all IEDS in each province. According to National Bureau of Statistics of China, Anhui had 17,616 units of enterprises above the designated size at the end of February, 2020. Those numbers in Hebei, Heilongjiang and Shandong are 13,020, 3,501, and 26,174, respectively. Table 1 lists all available RWP ratio and the corresponding date of each province. We focus on Anhui, Hebei, Heilongjiang, and Shandong provinces because we can collect data on at least 10 dates in these provinces. The RWP ratios of Anhui, Hebei, and Shandong provinces at the beginning of formal RWP are available. For Heilongjiang province, the data starts from one week later, which is February 17. Notice that the numerator used to calculate the RWP ratio actually refers to all enterprises that were in operation at the time, regardless whether they had experienced suspension or not. That is, the RWP ratio represents the sum of compartment *Q* and *B* corresponding to our model. Before official RWP, the ratio is less than 14% in Anhui and Hebei, and 21.8% in Shandong. On one hand, livelihood-enterprises keep their operation throughout the epidemic to provide essential supplies of electricity, gas, and water for residents. On the other hand, a large part of enterprises making medical material and daily necessities had already resumed work during the holiday to support epidemic control supplies and services. By mid March, the RWP ratio are nearly 100% in all provinces except for Heilongjiang, where the ratio is 92.4%.

**Table 1:** Data on resumption of work and production (RWP) of IEDS.

**Anhui**		**Hebei**		**Heilongjiang**		**Shandong**	
**Date**	**RWP ratio**	**Date**	**RWP ratio**	**Date**	**RWP ratio**	**Date**	**RWP ratio**
10/2/2020	0.138	10/2/2020	0.131	17/2/2020	0.369	9/2/2020	0.218
13/2/2020	0.277	16/2/2020	0.375	18/2/2020	0.39	10/2/2020	0.41
14/2/2020	0.319	19/2/2020	0.616	21/2/2020	43.1	11/2/2020	0.486
15/2/2020	0.367	20/2/2020	0.667	28/2/2020	0.64	12/2/2020	0.551
16/2/2020	0.404	21/2/2020	0.719	4/3/2020	0.73	14/2/2020	0.677
17/2/2020	0.461	24/2/2020	0.841	7/3/2020	0.808	15/2/2020	0.715
18/2/2020	0.515	25/2/2020	0.86	10/3/2020	0.868	17/2/2020	0.774
23/2/2020	0.827	26/2/2020	0.874	11/3/2020	0.885	18/2/2020	0.794
25/2/2020	0.92	29/2/2020	0.917	12/3/2020	0.91	19/2/2020	0.82
26/2/2020	0.948	1/3/2020	0.925	13/3/2020	0.917	20/2/2020	0.841
28/2/2020	0.974	3/3/2020	0.961	14/3/2020	0.924	21/2/2020	0.86
29/2/2020	0.981	8/3/2020	0.961			27/2/2020	0.98
		9/3/2020	0.979			1/3/2020	0.995
						10/3/2020	0.997

As mentioned above, the RWP ratio in our data is an aggregated count of compartment *Q* and *B*. To facilitate modeling, we assume that the size of *Q* stays constant (i.e., after formal RWP, no enterprises in normal operation at the time would go to suspension or permanent closure during our data period). To approximate the size of *Q*, we use the ratio of livelihood-enterprises among IEDS in each province in 2020^4^, which is 2.81%, 4.83%, 13.39%, and 4.83%, respectively, in Anhui, Hebei, Heilongjiang, and Shandong province. The assumption is reasonable since daily reported new cases have peaked in all four provinces before formal RWP, and government has pledged to spare no efforts to help companies resume production after Chinese New Year Holiday. Then, we subtract the ratio of livelihood-enterprises from RWP ratio to derive data on compartment *B*.

Due to data availability constraints, we do not possess data on the size of compartment *C*, which is the ratio of IEDS that close permanently during data period. To address this, we firstly collect record on newly established IEDS in 2020 for each province. Then, we calculate changes in the number of IEDS in 2020 using data of enterprise numbers at the end of 2019 and 2020. Changes in enterprise number also equal the result of using newly established enterprises number minus the number of enterprises that went down, which is the target information we need. Finally, we calculate the ratio of IEDS that closed in 2020 (Table 2). Since the data observation period is about 1 month, we divide the ratio of closure in a whole year by 12 as the approximate ratio of closure in 1 month.

**Table 2:** Ratio of IEDS that closed in 2020.

**Province**	**Number of new establishment**	**Number of change**	**Number of closure**	**Ratio of closure**
Anhui	2,174	987	1,187	$\frac{1,187}{17,616}=6.74\%$
Hebei	2,531	1,123	1,408	$\frac{1,408}{13,020}=10.81\%$
Heilongjiang	591	307	284	$\frac{284}{3,501}=8.11\%$
Shandong	6,679	4,086	2,593	$\frac{2,593}{26,174}=9.91\%$

## 4. Results

In this section, we firstly use data from the aforementioned four provinces (recall Anhui, Hebei, Heilongjiang, and Shandong) to calibrate the model's parameters. Then, we show several numerical simulations of “what-if” scenarios by varying the key parameters of the model and demonstrate the strength of this analytical framework by discussing several policy implications based on the tests.

### 4.1. Calibration of the model

We solve the ODE model in Python and calibrate the parameters β, γ, α, δ using the least square method. Matching the first day of data, February 9 is set as *T*_0_ for Shandong province. *T*_0_ of Anhui and Hebei is set to be February 10, and that of Heilongjiang is February 17. As mentioned earlier, *Q*(*T*_0_) is 2.81%, 4.83%, 13.39%, and 4.83% for Anhui, Hebei, Heilongjiang, and Shandong province, respectively. *B*(*T*_0_) equals RWP ratio on *T*_0_ minus *Q*(*T*_0_). We assume there was no permanent closure before formal RWP. That is, *C*(*T*_0_) is set to be zero. The assumption is reasonable since larger enterprises are better prepared to weather the negative shock from COVID-19. And we know from data that the number of IEDS that closed in 2020 is rather small. We also input the value of *C* at the end of our data period, which is the approximate ratio of closure in 1 month. Finally, *A*(*T*_0_) is 1−*Q*(*T*_0_)−*B*(*T*_0_)−*C*(*T*_0_). Table 3 shows the initial inputs of *Q*, *A*, *B*, and *C*.

**Table 3:** Initial inputs of *Q*, *A*, *B*, and *C*.

**Province**	***Q*(*T*_0_)**	***A*(*T*_0_)**	***B*(*T*_0_)**	***C*(*T*_0_)**
Anhui	0.028	0.862	0.110	0
Hebei	0.048	0.869	0.083	0
Heilongjiang	0.134	0.631	0.235	0
Shandong	0.048	0.782	0.170	0

Table 4 presents the optimized parameters β, γ, α, δ for each province. These parameters are derived from minimizing the least square loss function which consists of three parts: the squared sum of *Q*+*B* minus the RWP data value, the last-period value of *C* minus the approximated monthly closure ratio, and the last-period value of *Q* minus the livelihood-enterprises' ratio. The latter two parts of the loss function are further weighed by hyperparameters setting as 10.

**Table 4:** Optimized parameter values and growth rate of industrial value-added.

**Province**	**β**	**γ**	**α**	**δ**	**Growth rate of industrial value-added year on year**	
					**January and February 2020**	**March 2020**
Anhui	0.001	0	0.328	0.002	−12.1%	6.2%
Hebei	0	0	0.341	0.002	−9.4%	3%
Heilongjiang	0	0	0.151	0.001	−10.9%	−5.5%
Shandong	0	0	0.467	0.004	−10.6%	1.7%

The optimized value of parameters β (suspension rate) and γ (operation-to-closure rate) are zero in all four provinces, except that β = 0.001 in Anhui. Although the optimized suspension rate of Anhui is not zero, it is very small. These are in line with our assumption that no enterprises moved from normal operation to suspension or closure after formal RWP.

The optimized value of parameter α, which represents the rate at which enterprises move from suspension (*A*) to operation resumption (*B*), is 0.467 in Shandong, the largest among four provinces. It implies that IEDS resume operation faster in Shandong than the other three provinces. On February 4, 2020, Shandong was one of the first provinces in China to release a plan of resuming full production by the end of the month (48). According to data from the Department of Industry and Information Technology of Shandong province, as of February 6, 2020, 32 mask makers in the province have been running at full capacity. As a reference, the optimized value of parameter α is 0.341 in Hebei, 0.328 in Anhui, and 0.151 in Heilongjiang, the smallest among four provinces. Differences in resumption rates could also be partially explained by the differences in governments' policy responses to support the resumption of work and production. To connect with policies, we collect government policy documents in support of resumption of work and production from AMiner website. For each province, we calculate the number of policy documents issued by mid March, 2020 (see Table 5), and classified them into provincial and municipal levels. The government supportive policies mainly consist of operation support, tax relief, and deferral, liquidity measures, social security support, rent reduction, and so on. The Shandong government has issued 67 policy documents during February 1 and March 15, 2020, to support enterprises (see Table 5). By comparison, there were only 23 policies introduced during the same period in Heilongjiang, nearly one-third of the number of Shandong. The policies can be divided into provincial policies and municipal policies, which are launched by municipal governments and implemented only within a city. In Anhui, although the number of municipal policies is comparable with that of Shandong, there were deficiencies in provincial ones. Hebei has introduced 32 provincial policies, six more than the number in Shandong. However, municipal policies were scarce in Hebei during that time. We note that the estimated value of α is close between Anhui and Hebei. Anhui has launched more policy documents than Hebei, but the number of provincial policies in Hebei is almost twice that of Anhui.

**Table 5:** Number of government policy documents to support resumption of work and production by mid March, 2020.

**Province**	**Anhui**	**Hebei**	**Heilongjiang**	**Shandong**
Number of policy documents	61	40	23	67
Provincial level	17	32	21	26
Municipal level	44	8	2	41
Operation support	34	19	12	22
Tax	12	15	7	19
Finance	35	15	6	30
Social security	27	8	3	17
Rent reduction	13	8	2	13
Others	9	5	5	11

Finally, the optimized value of parameter δ is 0.02 in Anhui and Hebei, 0.01 in Heilongjiang, and 0.04 in Shandong. The optimized suspension-to-closure rates are small in all four provinces, which is in line with the fact that IEDS that closed during the outbreak accounted for only a small proportion.

To better understand the economic implications of the model parameters, we further look into industrial output data. Table 4 presents the growth rate of industrial value-added in the first three months of 2020. Put it together with the optimized parameter values, we can find that a larger suspension-to-closure rate is associated with a significant negative impact on industrial output. Intuitively, we would suppose that a larger resumption rate means enterprises experience a shorter disruption before quick recovery, which leads to a smaller economic loss. However, the cumulative added value of industries above designated size of January and February, 2020, decreased by about 11% year on year both in Shandong and Heilongjiang, although the optimized resumption rate of the former province is nearly three times faster than the latter. Another difference between these two provinces is that, the optimized suspension-to-closure rate of Heilongjiang is four times that of Shandong. In other words, the losses from a larger suspension-to-closure rate in Shandong is big enough to offset the benefits from a quicker resumption. Besides, while a large suspension-to-closure rate means an instant significant shock to industrial output, the negative effect of a slow resumption rate tends to persist longer. In March, the added value of industries above designated size increased by 1.7% on a year-on-year basis in Shandong. However, it continued to decrease in Heilongjiang.

**Figure 2:**
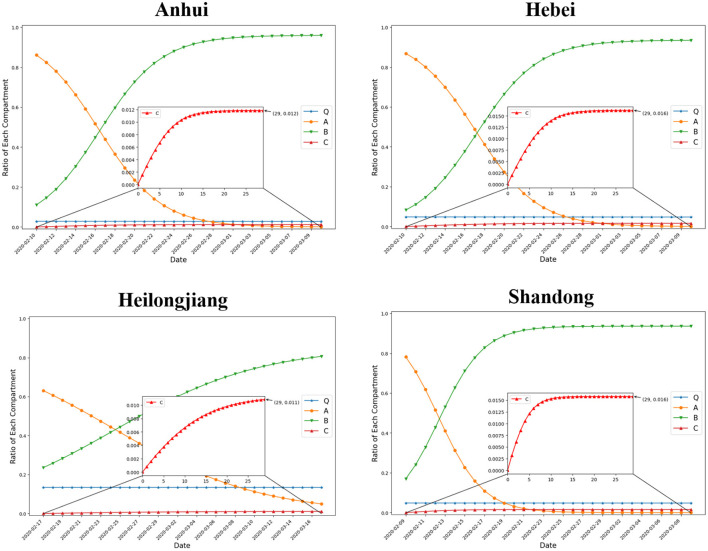
Dynamics of *Q*(*t*), *A*(*t*), *B*(*t*), *C*(*t*) over time.

Given *Q*(*T*_0_), *A*(*T*_0_), *B*(*T*_0_), *C*(*T*_0_), and the optimized value of parameters β, γ, α, δ, we graphically show the dynamics of *Q*(*t*), *A*(*t*), *B*(*t*), *C*(*t*) in Figure 2. At *T*_0_, the size of compartment *A* has passed its peak and is already in decline in all four provinces. It decreased fastest in Shandong, and slowest in Heilongjiang. The ratio of IEDS in suspension declined to below 1% at *t* = 14 in Shandong, and *t* = 22 in Anhui and Hebei. However, even at *t* = 29, there were 4.9% IEDS suspending operation in Heilongjiang. Then, we focus on changes in the size of compartment *B*, which monotonically increases with time. From the abovementioned, the size of *B* increased fastest in Shandong, and slowest in Heilongjiang. The ratio of IEDS that have resumed operation after suspension has risen to above 90% at *t* = 11 in Shandong, *t* = 15 in Anhui, and *t* = 18 in Hebei. However, there were only 80.6% IEDS that had resumed operation from suspension at *t* = 29 in Heilongjiang. The size of compartment *C* experiences a small increase in all provinces. These are enterprises that go down after a period of suspension.

Although we use China's RWP data to fit the model, two caveats should be well-noted here: Firstly, the COVID-19 outbreak in China overlapped with Chinese New Year, China's most popular nationwide holiday. On one hand, a great number of industrial enterprises would have suspended production during the holiday no matter of the epidemic. On the other hand, operation resumption were mostly arranged to start after the holiday, which was extended in 2020 due to COVID-19. Thus, in China, suspending production and resuming production did not happen simultaneously as our model suggested. Since we only have data on RWP, the process of enterprises moving from normal operation to suspension or closure can not be observed. Secondly, we do not have sufficient data to implement a precise fitting given the inclusion of four compartments in our model. To this end, we further conduct several numerical simulations to demonstrate the strength of the analytical models after the calibration.

### 4.2. “What-if” scenario simulation and policy implications

**Figure 3:**
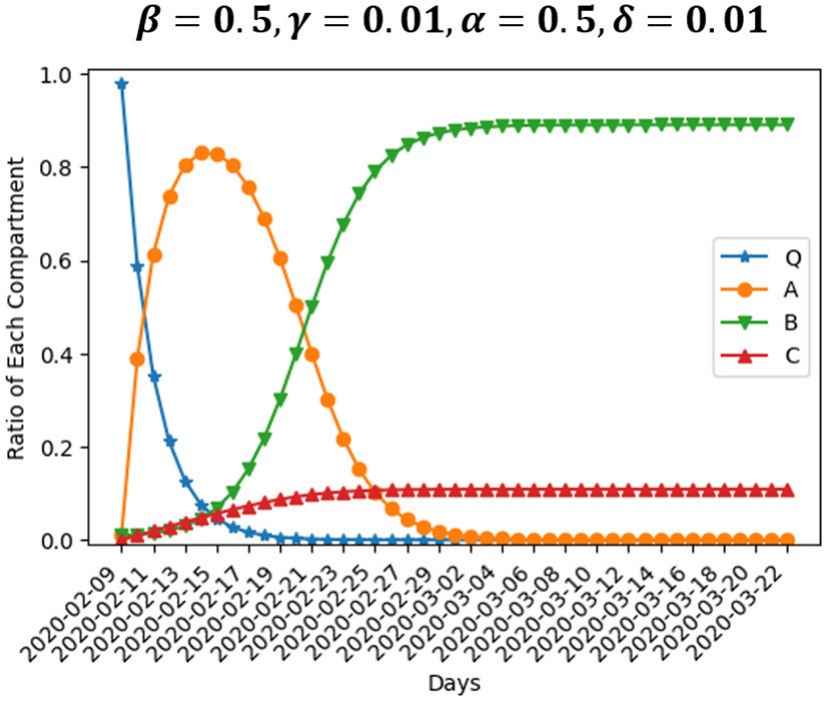
Baseline simulation.

Table 6 presents our baseline setting. We use *Q*(*T*_0_) = 0.98, *A*(*T*_0_) = *B*(*T*_0_) = 0.01, and *C*(*T*_0_) = 0 as initial value of compartment *Q*, *A*, *B*, and *C*. That is, most enterprises are in normal operation at *T*_0_. Parameters β, γ, α, and δ are set to be 0.5, 0.01, 0.5, and 0.01, respectively. Figure 3 graphically shows the dynamics of the size of four compartments within 43 days in baseline setting. The size of compartment *A* peaks at about Day 6, which means that the number of enterprises suspending operation starts to decline after that. In the end, 11% of enterprises close permanently and 89% resume operation after a period of suspension. We point out that the area size under curve *A*, which is the integral of the function of the curve, could be used to compare the loss from operation suspension under different scenarios. Similarly, the area size under curve *C* can be employed to analyze the differences in the loss from enterprise closure of each scenario.

**Table 6:** Baseline setting for simulation.

***Q*(*T*_0_)**	***A*(*T*_0_)**	***B*(*T*_0_)**	***C*(*T*_0_)**	**β**	**γ**	**α**	**δ**
0.98	0.01	0.01	0	0.5	0.01	0.5	0.01

**Figure 4:**
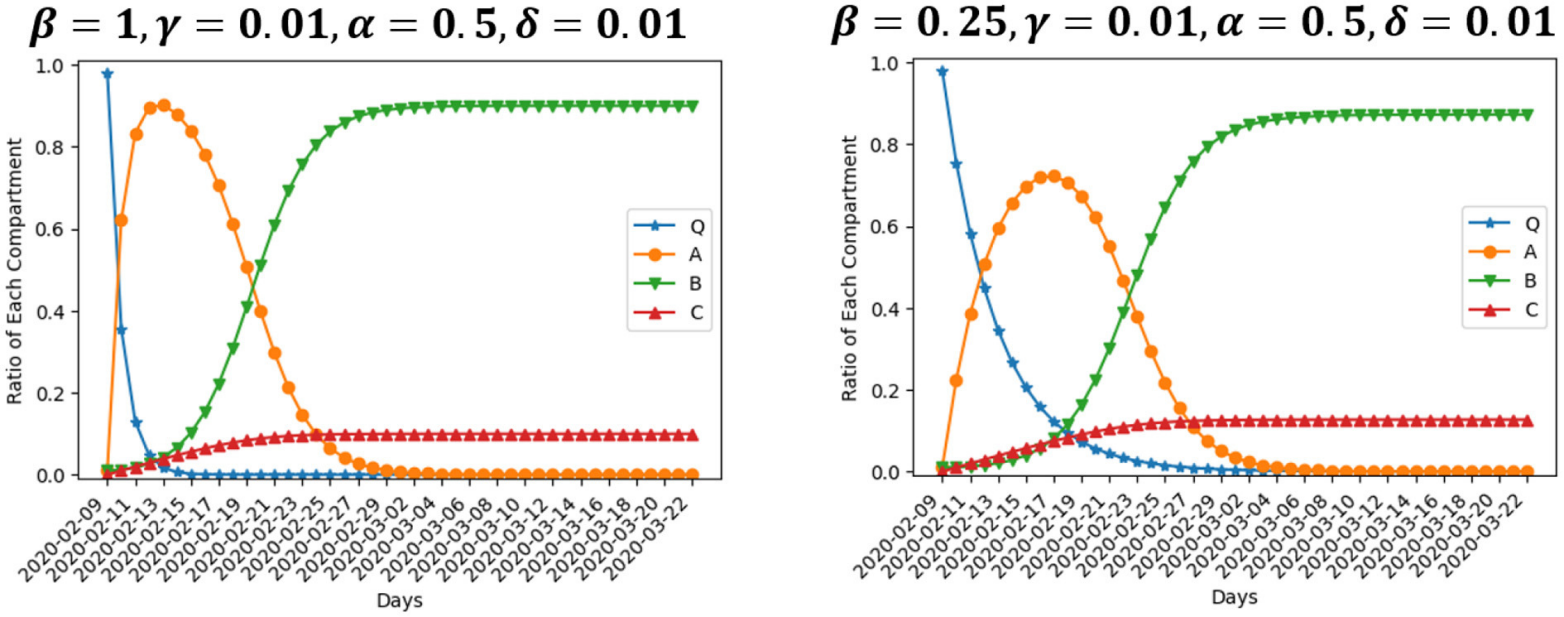
Simulation using different values of β.

We are interested in the evolution of the model when the value of parameters varies. Figure 4 presents two scenarios where β is set to be 1 and 0.25, respectively, while the rest of inputs are the same as our baseline setting. β is the rate at which enterprises move from normal operation to suspension. Large value of parameter β means operation suspension proceeds fast. The number of enterprises suspending operation peaks at about Day 5 if β = 1, while the peak arrives at about Day 9 if β = 0.25. Besides, the size of compartment *A* differs at the peak. The ratio of enterprises suspending operation at the peak is 90.2% in the former scenario, and 72.2% in the latter. This particular finding indicates that, changing the suspension rate from 1 to 0.25 could lead to fewer enterprises on average suffering from suspension. However, the enterprises that suspend operation would have to live with longer suspension. In the end, 10% of enterprises close permanently and 89% resume operation after a period of suspension if β = 1. The ratio of permanent closure and operation resumption in the end is 0.13 and 0.87, respectively, if β = 0.25, not much different from the scenario of β = 1. That is, policies aimed at depressing the suspension rate would barely reduce the negative effect of an outbreak.

**Figure 5:**
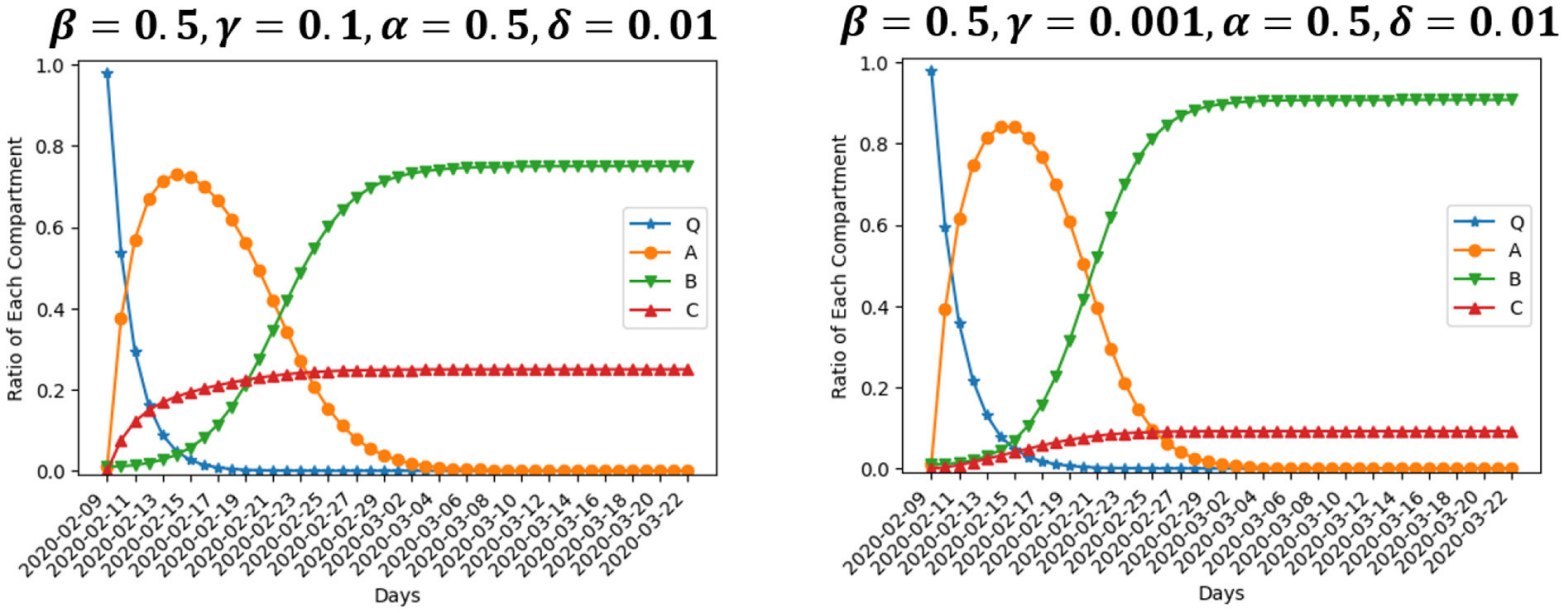
Simulation using different values of γ.

Figure 5 presents two scenarios where γ is set to be 0.1 and 0.001, respectively, while the rest of inputs are the same as our baseline setting. γ is the rate at which enterprises move from normal operation to permanent closure. Larger value of parameter γ means enterprises close at a faster rate. In the end, 25% of enterprises close permanently if γ = 0.1, while the ratio is 9% if γ = 0.001, nearly one-third of the former ratio. Enterprises experiencing suspension will recover and resume production, while enterprise closure leads to permanent loss of output. Changing the operation-to-closure rate from 0.1 to 0.001 reduces bankruptcy by nearly two-thirds, which could make a significant difference. Correspondingly, the ratio of enterprises resuming operation after a period of suspension is 75% in the former scenario, and 91% in the latter. The number of enterprises suspending operation peaks at about Day 6 in both scenarios. However, the number of suspended enterprises differs at the peak since more enterprises in closure means less in suspension. The ratio of enterprises suspending operation at the peak is 72.9% if γ = 0.1, and 84.2% if γ = 0.001. To conclude, the effect of a smaller γ is that more enterprises on average will experience suspension and fewer ones will end up with bankruptcy. Since the output loss to enterprise closure is larger than that to operation suspension, policies aimed at depressing the operation-to-closure rate could narrow the negative effect of an outbreak.

**Figure 6:**
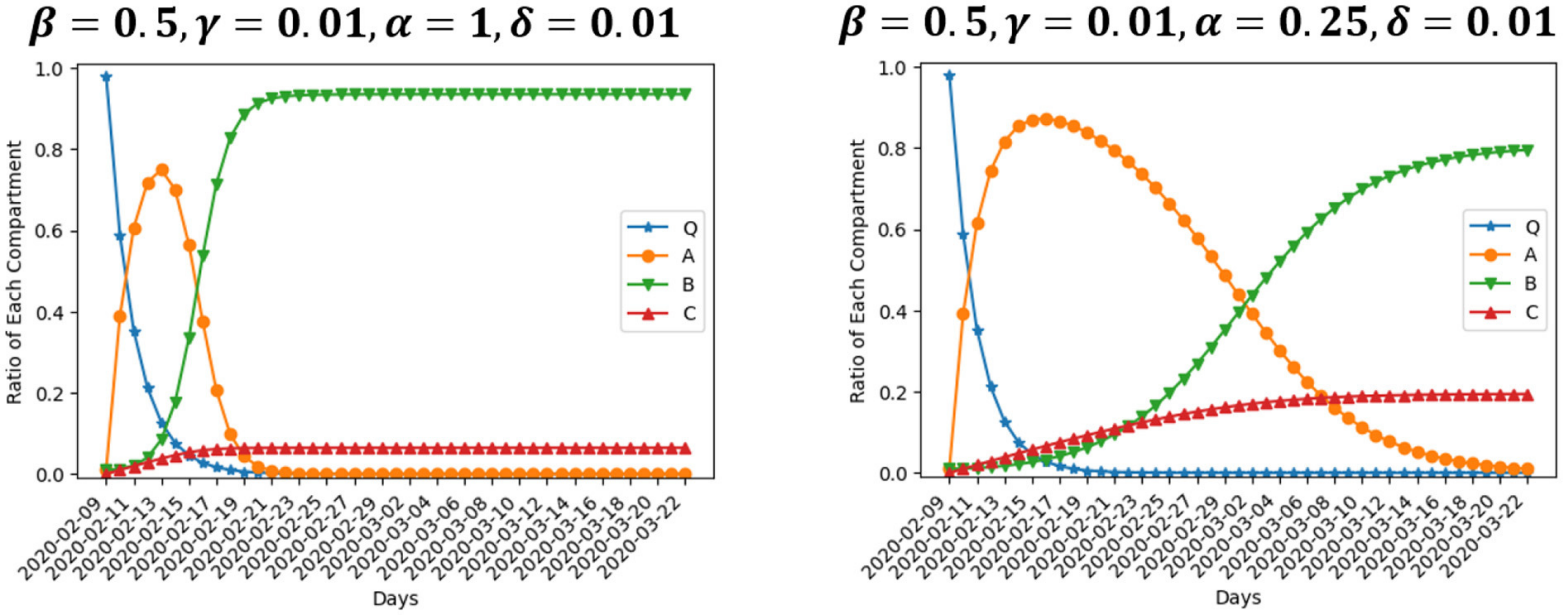
Simulation using different values of α.

Figure 6 presents two scenarios where α is set to be 1 and 0.25, respectively, while the rest of inputs are the same as our baseline setting. α is the rate at which enterprises move from suspension to operation resumption. Large value of α means enterprises experience a short suspension and resume operation quickly, while a small α means enterprises on average go through a long period of suspension. The size of compartment *A* declines slowly after it peaks at the value of 0.87 at about Day 8 if α = 0.25. If α = 1, it peaks at the value of 0.75 at about Day 5. This finding demonstrates that, changing the resumption rate from 0.25 to 1 would result in not only shorter suspension duration, but also fewer enterprises experiencing suspension on average. Besides, longer suspension leads to more closure. In this scenario, 19% of enterprises close permanently, 80% resume operation, and about 1% is still in suspension at Day 43, the end of our simulation period. However, the ratio of enterprises close permanently and resume operation in the end is 6% and 94%, respectively, if α = 1. Therefore, policies aimed at accelerating the resumption rate bring about significant benefits. Those policies could decrease the average number of enterprises experiencing suspension and shorten the suspension duration for those that do, as well as reducing the ultimate number of bankruptcies.

**Figure 7:**
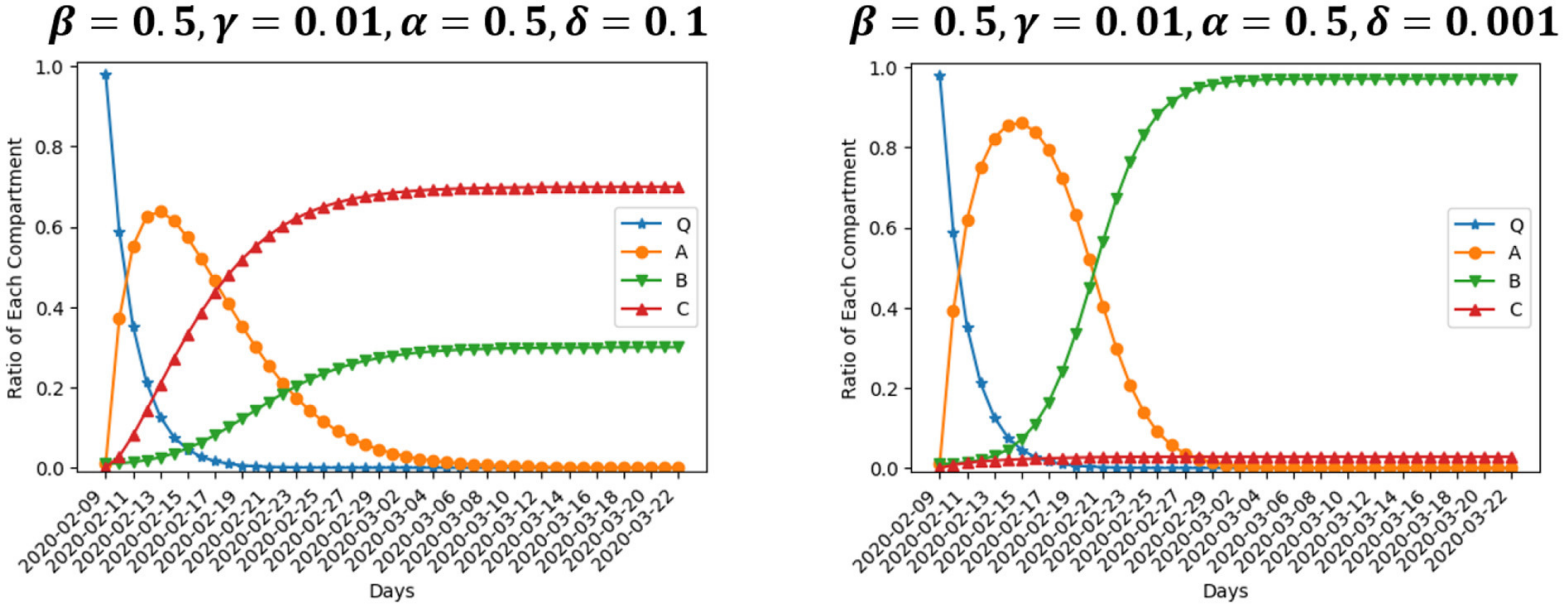
Simulation using different values of δ.

Figure 7 presents two scenarios where δ is set to be 0.1 and 0.001, respectively, while the rest of inputs are the same as our baseline setting. δ is the rate at which enterprises move from suspension to permanent closure. Larger value of parameter δ means enterprises close at a faster rate after a period of suspension, which also leads to a larger ratio of enterprises end up in permanent closure. If δ = 0.1, 70% of enterprises close permanently in the end, while only 30% survive the suspension. The ultimate ratio of permanent closure in this scenario is even much bigger than the one in the scenario of γ = 0.1 (Figure 5, left). If δ = 0.001, 3% of enterprises end up in permanent closure and 97% resume operation after a period of suspension, showing that, changing γ from 0.001 to 0.1 would result in almost 23 times more bankruptcies. It is important to for the government to depress the suspension-to-closure rate, and prevent enterprises in suspension from quick bankruptcy.

## 5. Conclusion

The COVID-19 pandemic has evolved beyond a public health crisis and caused severe economic consequences globally. During COVID-19, an enterprise may remain its normal operation throughout, though at a low chance. What's more likely is that it will experience temporary suspension out of epidemic control requirement, or even permanent closure for failure to tolerate chronic business loss. For those enterprises that sustain the pandemic and only suspend for a certain period, they will resume work and production when epidemic control and prevention conditions are satisfied and production and operation are adjusted correspondingly. In this paper, we develop a neat, yet representative, quantitative framework which contains a set of differential equations to capture such enterprises reactions against external pandemic shock over time, inspired by the susceptible-infectious-recovered (SIR) model. We collect data on IEDS resuming production from China's four provinces (Anhui, Hebei, Heilongjiang, and Shandong) spanning from February 9 2020 to mid-March 2020 to fit the model and calibrate the model by the least square method.

We found that the estimated value of parameter α, which represents the rate at which enterprises move from suspension to operation resumption, indicates that IEDS resume operation fastest in Shandong province, and slowest in Heilongjiang. This is consistent with the supportive policy intensity in four provinces. Shandong had issued 67 government documents introducing policies to support RWP by mid March, 2020. The number is the highest among four provinces, and nearly three times that of Heilongjiang. The estimated value of δ, which denotes the rate at which enterprises move from suspension to permanent closure is 0.004 in Shandong, 0.01 in Heilongjiang, and 0.02 in the rest two provinces. This reflects the fact that only a small proportion of IEDS close permanently under COVID-19, and a small increase in suspension-to-closure rate is associated with significant loss to industrial output, as in Shandong. We further perform theoretical simulations to verify the strength of the model. The results indicate that policies aimed at depressing the operation-to-closure rate narrow the negative effect of an outbreak. In contrast, policies aimed at accelerating the resumption rate bring significant benefits, whereas policies aimed at depressing the suspension rate would have little impact on the amount of economic loss to an outbreak. In addition, it is paramount for the government to depress the suspension-to-closure rate, and prevent enterprises in suspension from fast bankruptcy to save the economic meltdown.

Based on these findings, we suggest that governments' supportive policies toward enterprises under an epidemic should be primarily aimed at limiting bankruptcy. One important policy package to achieve the goal is providing access to finance, including measures such as credit payment deferral, interest payment suspension, debt rollover, access to new credit, and so on (49). Governments could also reduce taxes, defer tax payment or provide wage subsidies to ease the cost pressure of enterprises. Secondly, to accelerate production resumption, it is important for governments to assist enterprises to meet epidemic control and prevention requirements. Specifically, they could supply anti-epidemic materials, provide epidemic prevention instructions and support, ensure online government services, and so on.

The main contributions of this work are as follows. First, this study borrows ideas from the infectious disease community and novelly applies the SIR model to characterize the evolving state of enterprises affected by the COVID-19 pandemic. Second, we conduct a case study of four provinces in China to mimic the actual RWP process during the first wave of COVID-19 outbreak. Third, we conduct numerical simulations to examine the sensitivity of the model output to changes in parameter value, and provide policy implications on this basis.

Finally, we also acknowledge two limitations of this study. First, due to data availability issue, we cannot collect more detailed data to refine the simulation scenarios, which might have potential missing cases from our investigation and discussion. Second, the scope of this study was designed at a macroscopic scale in which four provinces in China were targeted. The idea of applying the proposed SIR-based analytical framework to enterprises' reaction toward COVID-19 can be plausibly extended to model individual agent's reaction to ubiquitous adverse events involving similar status at different phases (for instance, a detailed modeling of infrastructure components in response to natural disasters such as urban floods with incremental phases). Thus, to further test the applicability of the proposed framework the future work could focus on engaging more case studies and further develop extensions for the framework.

## Data availability statement

Publicly available datasets were analyzed in this study. This data can be found here: National Bureau of Statistics of China, http://www.stats.gov.cn/english/.

## Author contributions

HZ, ZH, and LX: conceptualization, methodology, data curation, validation, and software. HZ, ZH, LX, and JT: writing original draft, investigation, and writing review and editing. YC: funding and writing revision and editing. All authors contributed to the article and approved the submitted version.

## Footnotes

^1^According to National Bureau of Statistics of China, industrial enterprises consist of enterprises in the following sectors: mining and quarrying, manufacturing, and production and distribution of electricity, heat, gas, and water. Industrial enterprises above designated size are enterprise with annual revenue from principal business over 20 million yuan.

^2^According to their government announcements, the resumption of work and production should start no earlier than 24:00 on February 9.

^3^The main sources of data include official website of provincial government, Economic and Information Department, and Development and Reform Commission, and so on.

^4^As for Hebei, we do not get the ratio of livelihood-enterprises among industrial enterprises above designated size in 2020, and use the data in 2019 instead.
